# Actin-binding protein alpha-actinin 4 (ACTN4) is a transcriptional co-activator of RelA/p65 sub-unit of NF-kB

**DOI:** 10.18632/oncotarget.901

**Published:** 2013-02-28

**Authors:** Vasilisa Aksenova, Lidia Turoverova, Mikhail Khotin, Karl-Eric Magnusson, Eugene Tulchinsky, Gerry Melino, George P. Pinaev, Nickolai Barlev, Dmitri Tentler

**Affiliations:** ^1^ Institute of Cytology, Russian Academy of Sciences, Tikhoretsky av., 4, St. Petersburg, Russia; ^2^ Laboratory of Molecular Pharmacology, Saint-Petersburg Technological Institute, 26 Moskovsky Prospect, St. Petersburg, Russia; ^3^ Division of Medical Microbiology, Department of Clinical and Experimental Medicine, Linköping University, SE-581 85 Linköping, Sweden; ^4^ Department of Cancer Studies and Molecular Medicine, University of Leicester, RKCSB, LRI, Leicester, UK; ^5^ MRC Toxicology Unit, Leicester, UK; ^6^ Department of Biochemistry, University of Leicester, Lancaster Road, Leicester, UK

**Keywords:** ACTN4, RelA/p65, transcription regulation, MMP

## Abstract

ACTN4 is an actin-binding protein that participates in cytoskeleton organisation. It resides both in the cytoplasm and nucleus and physically associates with various transcription factors. Here, we describe an effect of ACTN4 expression on transcriptional activity of the RelA/p65 subunit of NF-kB. We demonstrate that ACTN4 enhances RelA/p65-dependant expression of c-*fos*, *MMP-3* and *MMP-1* genes, but it does not affect *TNC*, *ICAM1* and *FN1* expression. Importantly, actin-binding domains of ACTN4 are not critical for the nuclear translocation and co-activation of RelA/p65-dependent transcription. Collectively, our data suggest that in the nucleus, ACTN4 functions as a selective transcriptional co-activator of RelA/p65.

## INTRODUCTION

Alpha-actinin 4 (ACTN4) is an actin-binding protein which belongs to the spectrin superfamily. It predominantly localises to the cytoplasm and focal adhesion contacts. Primarily, ACTN4 was found to participate in the cytoskeleton organisation, cytokinesis, regulating cell adhesion and shape [1, 2]. Being a part of cytoskeleton, ACTN4 plays a crucial role in remodelling of actin cytoskeleton and in formation of protrusions, which potentiate migration of normal and cancer cells [3].

According to the domain structure, ACTN4 can be subdivided into three major regions. The N-terminal region includes two calponin homology domains (CH1-2). Due to the actin-binding domain (ABD) located in this region, ACTN4 directly cross-links the actin filaments and drives them to focal adhesion sites via adhesion plaque proteins [4, 5]. The central part of ACTN4 consists of four spectrin repeats (SR1-4), which are responsible for dimerisation in an anti-parallel manner; they also participate to the assembly of multiprotein structures involved both in the cytoskeleton architecture and signal transduction [6]. Finally, together with the C-terminus, spectrin repeats mediate interactions with a wide group of cytoskeleton and non-cytoskeleton proteins such as vinculin, MEKK1 kinase, MAGI, BP-180 [7, 8, 9]. The C-terminus region of ACTN4 includes two EF hand domains (EF1-2) and the calmodulin-homology domain [10].

A nuclear localisation of ACTN4 has been reported [1, 11], as well as its interaction with a number of nuclear proteins like NF-Y [12], DNaseY [13], RelA/p65 subunit of the nuclear factor-κB (NF-κB) [14], heterogeneous nuclear ribonucleoproteins [15] and HDAC7 [16]. ACTN4 may therefore participate in regulation of transcription. Accordingly, ACTN4 modulates transcriptional activity of estrogen receptor, retinoic acid receptor and peroxisome proliferator-activated receptor gamma [17, 18]. Nevertheless, its nuclear functions are not fully understood, and the precise mechanisms of its nuclear translocation have not been revealed yet.

The NF-kB family includes several members, RelA, c-Rel, RelB, p50 and p52, all able to regulate, as transcription factors, a number of molecules involved in cancer [19, 20, 21, 22, 23], cellular senescence [24], hypertonic stress [25], immunity [26] as well as host defence against pathogens [27, 28]. A major function of NF-κB occurs in the innate and adoptive immunity [29] is mediated by its ability to regulate cytokines [30], chemokines [31], and adhesion molecules [32]. However, there are still several open questions related to the selective functions of individual NF-κB family members [33] during a coordinated cellular response to pathogens, and the related molecular mechanisms. The effect on autophagy [34], TNF [35, 36, 37, 38, 39], TRAIL [40, 41] is still under intense investigation [42, 43], and in particular several interactions seems able to fine tuning NF-κB function, including for example regulators of histone acetylation [44], ubiquitin [45] and IAPs [46]. To this end, a potential regulation by ACTN4 could be of particular interest.

To further elucidate ACTN4 role in nuclear processes, we investigated functional significance of its interaction with RelA/p65 subunit of transcriptional factor NF-kB. We have found that ACTN4 can modulate transcriptional activity of RelA/p65. This function does not directly depend on its ability to bind actin or to form dimers. Nevertheless, ACTN4 regions responsible for actin binding and dimerisation are required for its nuclear translocation. Our data indicate that ACTN4 may co-regulate expression of certain genes together with transcription factors such as NF-kB.

## RESULTS

### ACTN4 does not influence on nuclear accumulation of RelA/p65

**Figure 1:**
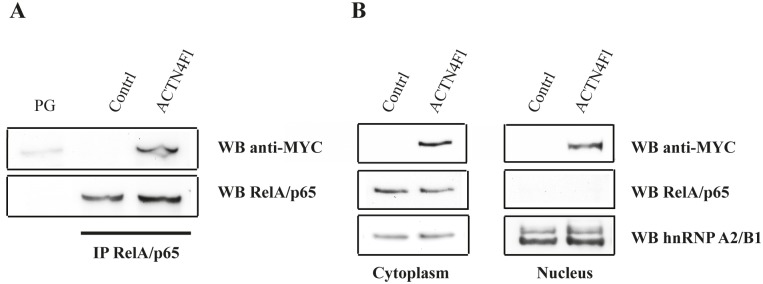
Over-expressed ACTN4 co-immunoprecipitates with RelA/p65 in the cytoplasm of HEK293T cells but does not mediate RelA/p65 nuclear translocation (A) RelA/p65 was immunoprecipitated from cytoplasm protein extracts of HEK293T cells with RelA/p65-specific antibody. Bound proteins were separated by SDS-PAGE followed by immunoblotting with anti-MYC and anti-RelA/p65 antibodies. No RelA/p65 specific signal was detected after incubation of cytoplasm extracts with empty Protein G Sepharose beads (PG lane). (B) Western blot analysis of cytoplasm and nuclear protein extracts using MYC-, RelA/p65- and hnRNPA2/B1-specific antibodies. hnRNPA2/B1 was used as a loading control.

The transcription factor NF-kB is sequestered in the cytoplasm of most cells by specific inhibitory protein IkBα [47]. Nuclear translocation of NF-kB is required for its full activation and ability to activate gene expression. In fact, RelA/p65 subunit of NF-kB co-immunoprecipitates with ACTN4 and localises along stress fibres in A431 cells [14, 48]. To study the interaction between ACTN4 and RelA/p65 and their co-localisation in HEK293T cells, we prepared a construct, expressing full-length ACTN4 (ACTN4Fl) with MYC-tag at the N-terminus. Co-immunoprecipitation shows that ACTN4Fl is present in complexes with RelA/p65 in cytoplasm of HEK293T cells (Fig.1 A). Since ACTN4 interacts with RelA/p65 both in the cytoplasm and nucleus, we evaluated the ability of ACTN4 over-expression to affect the RelA/p65 nuclear accumulation. Immunoblotting of nuclear and cytoplasm protein extracts demonstrates that ACTN4Fl over-expression alone does not lead to nuclear accumulation of RelA/p65 in comparison to control cells (Fig.1 B).

### ACTN4 co-activates RelA/p65-dependant expression of c-fos and MMP-3 genes

**Figure 2:**
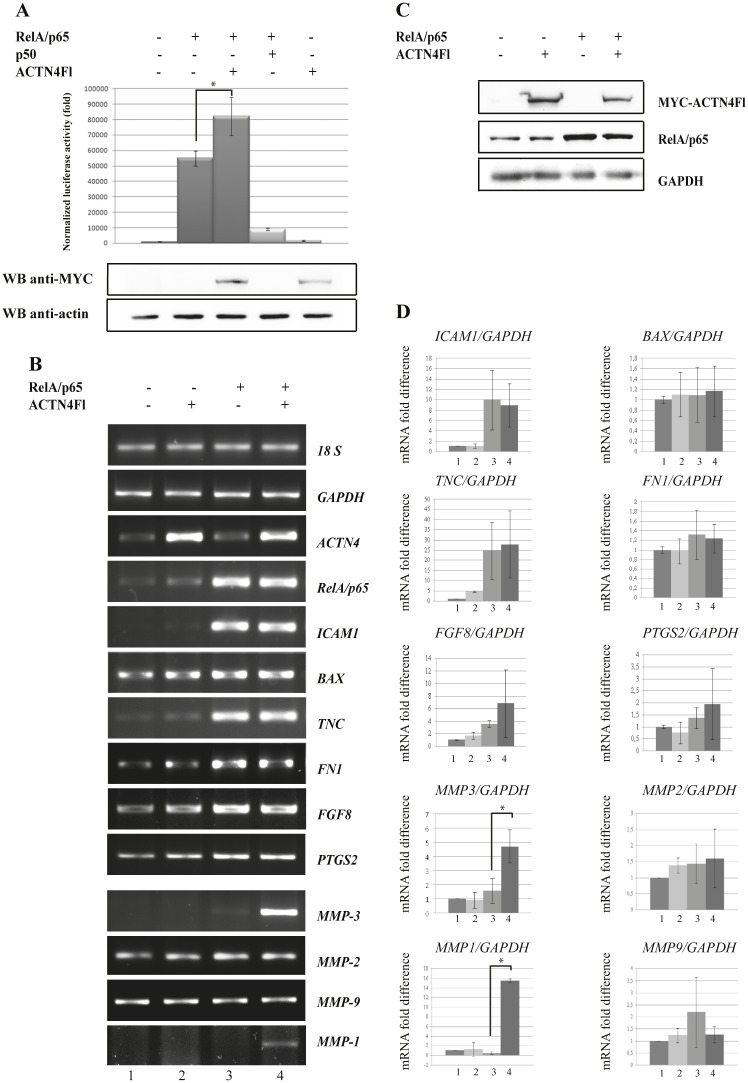
Co-expression of ACTN4Fl and RelA/p65 leads to enhanced expression of RelA/p65-regulated genes (A) A431 cells were transfected with luciferase reporter vector containing luciferase gene under *c-fos* mouse promoter, and expression plasmids with *ACTN4Fl*, *RelA/p65* and *p50*. The ratios of luciferase-to-β-galactosidase in comparison to an empty luciferase construct are presented. Expression of exogenous ACTN4 (the lower panel) was determined by Western blot analysis using anti-MYC antibody (*P< 0.02 for ACTN4Fl+RelA/p65 versus RelA/p65). (B) ACTN4Fl influence on transcription activity of RelA/p65 subunit of transcription factor NF-kB in regard to *MMP-3* gene promoter. HEK293T cells were transfected with plasmids coding *ACTN4Fl* and *RelA/p65*. Over-expression of RelA/p65 alone noticeably induces expression of *TNC* and *ICAM1* genes, but does not have significant effect on expression of *BAX*, *FGF8*, *PTGS2*, and *FN1* genes. The lower panel represents RT-PCR of MMPs: *MMP-2*, *MMP-9*, *MMP-1* and *MMP-3* genes. (C) Western blot analysis of ACTN4Fl and RelA/p65 expression in HEK293T cells. (D) Quantification data of RelA/p65 regulated genes after transfection of RelA/p65 and ACTN4Fl. The data show fold difference of mRNA level of RelA/p65-regulated genes in regard to *GAPDH* gene. HEK293T cells were transfected either with ACTN4Fl (lanes 2), or RelA/p65 (lanes 3), or co-transfected with ACTN4 and RelA/p65 (lanes 4). Non-transfected HEK293T cells is shown at lanes 1. Co-expression of RelA/p65 with ACTN4Fl leads to strong increase of *MMP-3* gene expression (*P<0.05 - *MMP-3* and *P<0.02 - *MMP-1* for ACTN4Fl+RelA/p65 versus RelA/p65). The data represent an average of at least three independent experiments, ±SD.

In order to further evaluate a possible influence of ACTN4 on RelA/p65-dependant gene expression, we employed a luciferase transcription assay. The pfLUC reporter construct that contained the luciferase gene under control of the minimal (-59 to +109) *c-fos* promoter [49] along with *ACTN4* and *RelA/p65* were transiently transfected into A431 cells. We have found that over-expression of full-length ACTN4 alone does not activate transcription from *c-fos* promoter (Fig.2 A). However, co-expression of ACTN4Fl with RelA/p65 enhances luciferase activity versus RelA/p65 alone (Fig.2 A). These data indicate that ACTN4Fl specifically co-activates the RelA/p65 subunit of transcription factor NF-kB, and can potentially regulate expression of other RelA/p65-dependent genes. To determine whether this regulation is gene-specific or it is a more general phenomenon, we analysed expression of seven additional genes known to contain RelA/p65 binding site in the promoter. HEK293T cells were transiently transfected with plasmids that express ACTN4Fl and RelA/p65 followed by a semi-quantitative RT-PCR analysis of *TNC*, *ICAM1*, *FGF8*, *BAX*, *PTGS2*, *FN1* and *MMP-3* gene expression (Fig.2 B, D). Over-expression of RelA/p65 alone noticeably induces expression of *TNC* and *ICAM1* genes, but does not effect on expression of *BAX*, *FGF8*, *PTGS2*, and *FN1* genes. Moreover, none of these genes is affected by over-expression of ACTN4Fl. However, co-expression of RelA/p65 with ACTN4Fl significantly enhances expression of *MMP-3* gene (Fig.2 B lower panel and D). These results indicate that ACTN4Fl can regulate *MMP-3* gene expression in a RelA/p65-dependant manner. All together, our data demonstrate that ACTN4 can be a co-activator of RelA/p65, even though not all genes display this effect.

### ACTN4 affects matrix metalloproteinases gene expression

Matrix metalloproteinases (MMPs) belong to a large family of endopeptidases that cleave a wide range of substrates including numerous matrix proteins, growth factors and proteases [50]. MMP-3 plays a major role in remodelling of collagens, and shows substrate specificity to several other matrix proteins and proteoglycans [51]. Besides *MMP-3*, NF-kB can activate other *MMP* genes, including *MMP-1*, *MMP-9* and *MMP-2* [52, 53]. We examined whether ACTN4 might also activate expression of these genes. *MMP-2* and *MMP-9* genes do not exhibit any changes in their mRNA levels in response to ACTN4Fl and RelA/p65 over-expression (Fig.2 B lower panel and D). However, the mRNA level of the *MMP-1* gene is affected by ACTN4Fl and RelA/p65 (Fig.2 B lower panel and D). These data show that other MMPs, such as *MMP-1*, can be regulated in a similar manner to *MMP-3*.

### Dimerisation and binding to F-actin are not required for the nuclear import and functioning of ACTN4

**Figure 3:**
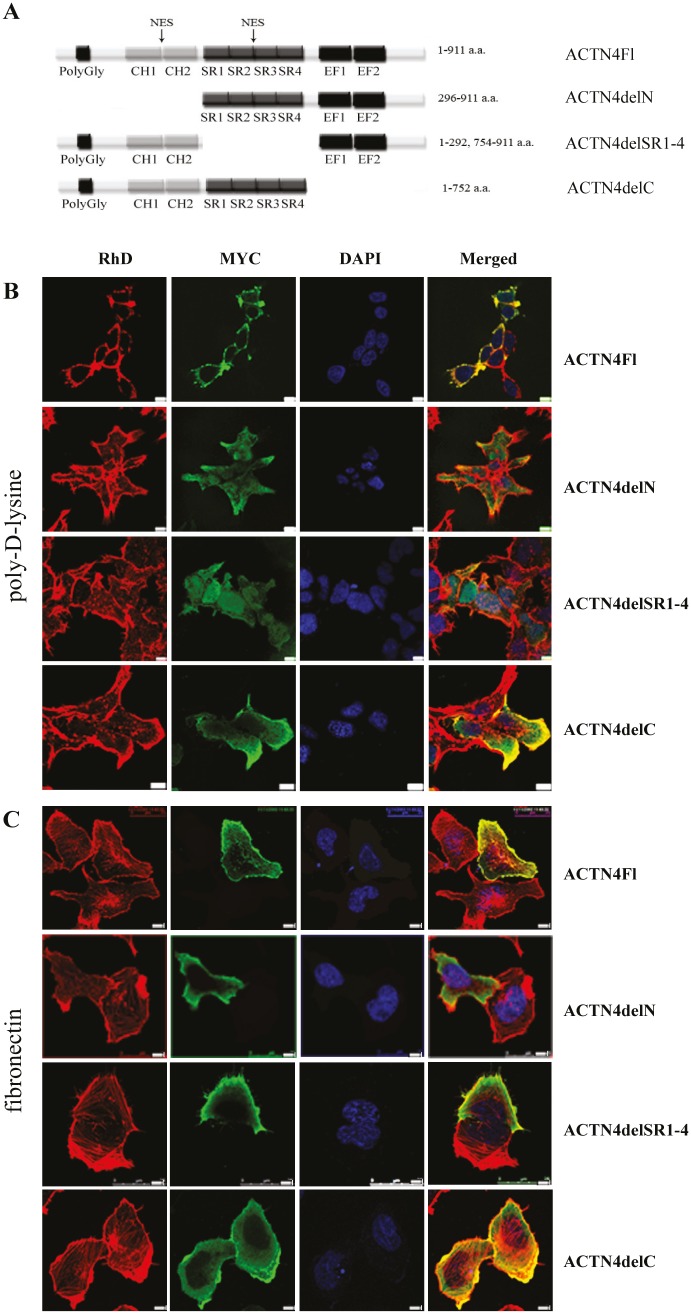
Intracellular distribution of the ACTN4 deletion mutants in transfected HEK293T cells (A) Scheme of ACTN4 deletion variants with MYC-tag on N-terminus. (B) HEK293T cells were transfected with expression plasmids coding ACTN4Fl, ACTN4delN, ACTN4delSR1-4, ACTN4delC, and cultured on poly-D-lysine coated cover slips. (C) HEK293T cells spread on EMC protein (fibronectin). Bar scale is 5 um.

The main functions of ACTN4 in the cytoplasm are mediated by its ability to form homodimers and bind F-actin filaments. Similarly, dimerisation and actin-binding may be critical for translocation of ACTN4 into the nucleus and its function as a co-regulator of RelA/p65. To test this hypothesis, we constructed three expression plasmids, containing ACTN4 with deletions of the N-terminal region with actin-binding domain, the C-terminal region, and SR1-4 domains. All deletion variants contained a MYC-tag at the N-terminus that allowed visualising exogenous protein (Fig.3 A).

To determine the intracellular distribution of ACTN4 deletion variants, we carried out transfection of HEK293T cells followed by immunofluorescence using anti-MYC-tag antibodies. Transfected HEK293T cells were spread on cover slips covered with poly-D-lysine for 24 hours. The subcellular distribution of ACTN4 compared to the actin cytoskeleton and the nucleus was obtained by staining of corresponding structures with rodamin-phalloidin (RhD) and DAPI, respectively (Fig.3). Analysis of the localisation of exogenous protein variants shows that the deletion of the N-terminal and SR regions leads to nuclear accumulation of ACTN4 along with disruption of interaction with structures of the actin cytoskeleton (Fig.3 B). The localisation of protein variant with the deletion of C-terminal part of ACTN4 is very similar to the intracellular localisation of ACTN4Fl with the exception of diffused distribution through the cytoplasm along with binding to actin structures (Fig.3 B). These results are in keeping with a previous report, showing the presence of two nuclear export signals in ACTN4 sequence inside calponin-homology and SR domains [11].

Next, we decided to test whether ACTN4 localisation changes in response to stimuli from extracellular matrix (ECM). Thus, we spread transfected HEK293T cells on fibronectin. The cell spreading on fibronectin leads to a predominantly cytoplasmic localisation of the ACTN4delN and ACTN4delSR1-4 deletion mutants (Fig.3 C). This intracellular distribution is opposite to the one observed in cells cultured on poly-D-lysine. These protein variants do not co-localise along the filaments of actin cytoskeleton, but, at the same time, they predominantly localise outside the nucleus. The localisation of ACTN4Fl and ACTN4delC on fibronectin is very similar to that observed on poly-D-lysine. The ACTN4delC protein variant is predominantly localised in cytoplasm except weaker co-localisation with stress-fibres. Taking together, these data demonstrate that the N-terminal and SR domains are critical for the nuclear/cytoplasmic distribution of ACTN4 and F-actin binding in response to ECM cell signalling.

**Figure 4:**
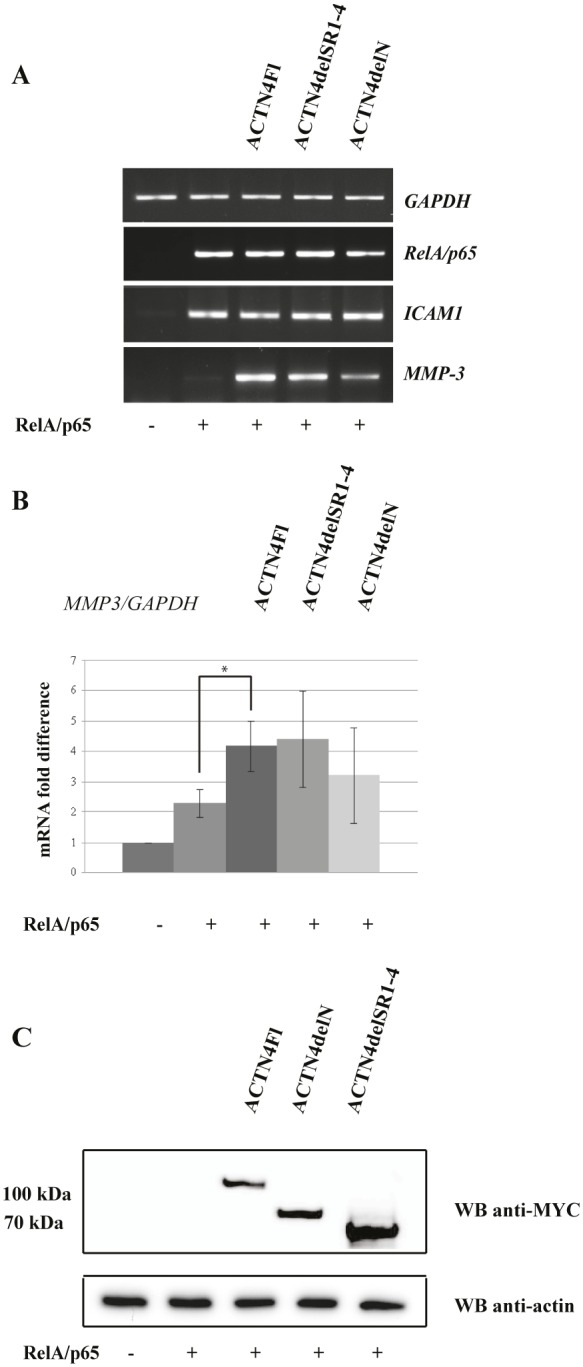
Over-expression of ACTN4delN and ACTN4delSR1-4 enhances activation of *MMP-3* expression by RelA/p65 (A) The RT-PCR analysis of *GAPDH*, *RelA/p65*, *MMP-3*, *ICAM1* expression level in 67 hours after transfection of HEK293T cells with ACTN4 deletion variants and/or RelAp65. (B) Quantification data of RelA/p65 and ACTN4 deletion variants co-expression. The data represent average fold difference of *MMP-3* mRNA level to *GAPDH* mRNA level of at least three independent experiments, ±SD. (*P<0.01 for *MMP-3* for ACTN4Fl+RelA/p65 versus RelA/p65) (C) Western blot analysis of ACTN4 mutants expressed in HEK293T cells using MYC and actin specific antibodies.

Considering the abovementioned data, we next examined the significance of ACTN4 dimerisation for its ability to activate *MMP-3* gene expression. HEK293T cells were transiently transfected with ACTN4delN, ACTN4delSR1-4 and RelA/p65 expression plasmids followed by the analysis of *MMP-3* expression level. Western blot analysis of cellular lysates confirms the expression of MYC-tagged ACTN4 deletion variants in HEK293T cells (Fig.4 C). We have found that variants with deletions of the spectrin or N-terminal domains activate *MMP-3* gene expression in the presence of RelA/p65 (Fig.4 A, B), however to a slightly less extent than the full-length ACTN4 protein. These results indicate that ACTN4 domains responsible for binding to F-actin and homodimerisation are not critical for co-activation of RelA/65 in respect to *MMP-3* gene expression.

## DISCUSSION

In the present study, we have demonstrate that ectopic expression of ACTN4 with the RelA/p65 subunit of transcription factor NF-kB, but not ACTN4 alone, potentiates transactivation of the mouse *c-fos* gene by RelA/p65. In addition, ACTN4 co-activates RelA/p65-mediated gene expression of the matrix metalloproteinases *MMP-3* and *MMP-1* genes, but does not affect expression of other RelA/p65 target genes, including *BAX*, *TNC*, *FGF8*, *PTGS2*, *ICAM1* and *FN1*. Moreover, we demonstrate that ACTN4 does not potentiate nuclear accumulation of RelA/p65. Alteration of NF-kB activity via post-translational modifications without inducing nuclear translocation has been demonstrated [54, 55, 56, 57]. Apparently, ACTN4 co-activates RelA/p65-dependent transcription by an unknown mechanism.

It has previously been shown that ACTN4 can be a co-activator of nuclear receptors like ER alpha, RAR, PPAR gamma [17, 18] and transcription factor MEF2 [16]. Along with these observations, our data demonstrate that ACTN4 can be a co-activator/positive regulator for various transcription factors, including RelA/p65. It is quite possible that a common mechanism, which is yet to be uncovered, is involved in all these cases. In addition, we have shown that only *MMP-3* and *MMP-1*, but not *MMP-2* and *MMP-9* are directly regulated by ACTN4. Similarly, such regulation of different subtypes of MMPs was also observed in recent studies on the impact of oxidative stress and heat shock on cells [51, 58]. The fact that ACTN4 acts as a RelA/p65 co-activator in respect to some genes but not to the others suggests that the requirement in ACTN4 may be promoter-specific.

Nuclear function of ACTN4 as a transcriptional co-activator is likely associated with its cellular distribution. In particular, this effect has been demonstrated for regulation of the *Fshb* gene via gonadotropin-releasing hormone [59]. Translocation of ACTN4 into the nucleus occurs in spite of an absence of a classic nuclear localisation signal. Recently, two signals of ACTN4 nuclear export were determined in the middle part of calponin-homology domains and spectrin regions. It has been proposed that nuclear import of ACTN4 can be mediated by direct interaction of spectrin region with the nuclear pore complex [11]. Here, we analysed the intracellular distribution of ACTN4 with deletions of the amino-, carboxyl- and SR domains in cells cultured either on poly-D-lysine or on fibronectin. Interestingly, all ACTN4 deletion mutants retained their ability to enter the nucleus in cells on poly-D-lysine. These data indicate that the SR-dependent nuclear import may be not the only mechanism mediating ACTN4 nuclear translocation. Consequently, ACTN4 may contain more than one sequence responsible for nuclear import. Moreover, nuclear accumulation of ACTN4delN and ACTN4delSR1-4 mutants may suggest that these regions contain sequences that keep ACTN4 in the cytoplasm, probably due to their involvement into F-actin binding.

Our immunofluorescence data showed that ACTN4delN and ACTN4delSR1-4 variants displayed nuclear localisation. Therefore, these mutants were selected for analysis of their ability to activate *MMP-3* gene expression. According to our results, both protein variants are able to co-activate the *MMP-3* gene expression, although to a lesser extent in comparison to the full-length ACTN4 protein. These data also suggest that dimerisation of ACTN4 and its binding to nuclear actin are not critical for RelA/p65-mediated gene expression. Thus, we speculate that the nuclear role of ACTN4 in regulation of transcription factor activity differs from its function in the cytoplasm and does not depend on the actin binding.

## MATERIALS AND METHODS

### Cell cultures and antibodies

A431 and HEK293T cell lines (Cell Culture Collection, Institute of Cytology, Russian Academy of Sciences) were cultured in complete Dulbecco’s Modified Eagle’s Medium (DMEM, Gibco) supplemented with 10% fetal bovine serum (FBS, Sigma) in 5% CO_2_ at 37°C.

The following antibodies were used in this study: anti-MYC-tag (M4439, Sigma), anti-RelA/p65 (sc-372 and sc-8008 AC, Santa Cruz), anti-actin (AC-17, Sigma), anti-hnRNP A2/B1 (R4653, Sigma), anti-GAPDH (2118, Cell Signaling), Alexa 488-conjugated anti-mouse antibodies (A21200, Invitrogen), HRP-conjugated anti-mouse antibodies (A9044, Sigma).

### Plasmid constructions

Total RNA was purified from A431 cells using TRIzol Reagent (Invitrogen). The cDNA synthesis was performed by RevertAid – First Strand cDNA Synthesis Kit (Fermentas) according to manufacturer’s protocol. Full length *ACTN4* cDNA (*ACTN4Fl*, 1-2736 nt from start codon) and deletion variants were amplified with primers listed in Table 1 by Pfu DNA-polymerase (Fermentas), and cloned into pCS2MT expression vector. ACTN4 deletion variants were constructed by PCR from *ACTN4Fl* cDNA: deletion of spectrin-homology domains (ACTN4delSR1-4, 293-753 a.a.); deletion of EF-hand domains (ACTN4delC, 753-911, a.a.); deletion of N-terminus, including ABD (ACTN4delN, 1-295 a.a.).

**Table 1:** Oligonucleotides used for generation of ACTN4 deletion variants

ACTN4 variant	Primer sequence (5’→3’)
ACTN4Fl	Forward GGAATTCTATGGTGGACTACCACGCGGCG Reverse GCTCTAGATCACAGGTCGCTCTCGCCATACAAG
ACTN4delSR1-4	Forward CAGATCCTCACCCGCGACGCCAAG Reverse CTCGTTCTCTTGGTTGACAGCCAGCACC
ACTN4delN	Forward GGAATTCGATGGAGGACTACGAGAAGCTGGC Reverse GCTCTAGATCACAGGTCGCTCTCGCCATACAAG
ACTN4delC	Forward GGAATTCTATGGTGGACTACCACGCGGCG Reverse GCTCTAGATCAGTTCTCCACCTCGTTGATGGTGC

### RT-PCR

Expression of RelA/p65-dependent genes was established by semi-quantitative polymerase chain reaction. cDNA was generated from 2.5 ug of total RNA using RevertAid – First Strand cDNA Synthesis Kit (Fermentas). PCR was performed in 20 ul reaction volume containing 2 ul of cDNA, 1 pmol of forward and reverse primers, 2 mM MgCl_2_, and 0.2 U Taq-polymerase (Fermentas). The primers used for semi-quantitative PCR are listed in Table 2.

**Table 2:** Oligonucleotides used for amplification of cDNAs

Gene	Primer sequence (5’→3’)
	Forward Reverse
GAPDH	CCATCTTCCAGGAGCGAGA GGCAGTGATGGCATGGACTGT
18S	AAACGGCTACCACATCCAAG CAATTACAGGGCCTCGAAAG
RelA/p65	CGAATGGCTCGTCTGTAGTG TGGTGGTATCTGTGCTCCTC
ACTN4	GGGCAGAAGAGATTGTGGAC TTGTTCAGGTTGGTGACAGG
ICAM1	CACAGTCACCTATGGCAACG CTGAGACCTCTGGCTTCGTC
TNC	CTCTGGTGCTGAACGAACTG GGAAACTGTGAACCCGTAGG
FN1	CTACCAAGGCTGGATGATGG TGTGCCTCTCACACTTCCAC
BAX	CATGTTTTCTGACGGCAACT GGAGGAAGTCCAATGTCCAG
PTGS2	TGGCTACAAAAGCTGGGAAG AACTGATGCGTCAAGTGCTG
FGF8	GGACACCTTTGGAAGCAGAG CCCTCGTACTTGGCATTCTG
MMP-3	TGCTTTGTCCTTTGATGCTG CCAGCTCGTACCTCATTTCC
MMP-9	GCCAGTCCACCCTTGTGCTCTT TCGGGCAGGGACAGTTGCTT
MMP-1	TTCGGGGAGAAGTGATGTTC TCCTTGGGGTATCCGTGTAG
MMP-2	GCTCAGATCCGTGGTGAGAT GGTGCTGGCTGAGTAGATCC

RT-PCR quantification was done by densitometry analysis of Quantity One 1-D software. Statistical comparisons were performed using Student’s *t*-test of at least three independent experiments.

### Immunofluorescence

HEK293T cells were transfected with expression plasmids coding full-length ACTN4 (ACTN4Fl) and deletion variants (ACTN4delSR1-4, ACTN4delN, ACTN4delC) with Turbofect reagent (Fermentas). Cover slips for cell spreading were covered with poly-D-lysine hydro bromide (Sigma) or fibronectin (Fluka) according to manufacturer’s protocol. Following 24 hours after transfection, HEK293T cells were spread on poly-D-lysine for 24 hours or fibronectin for 1 hour. Cells were fixed with 4% paraformaldehyde in PBS at room temperature for 15 minutes, and permeabilized in 0.1% Triton X-100 for 6 minutes. Protein intracellular localisation was detected by specific primary antibodies to MYC-tag and secondary Alexa 488-conjugated anti-mouse antibodies. The cytoskeleton and nucleus were visualised with rhodamine-phalloidine (Invitrogen) and 4,6-diamidino-2-phenylindole (DAPI, Sigma), respectively. Images were acquired using confocal microscope Leica TCS SP5.

### Luciferase assay

A431 cells were seed in 6*10^4^ on 24-well plates a day before transfection. Transfection was performed using Lipofectamine 2000 (Invitrogen) according to manufacturer’s instruction. Total amount of DNA was 3 ug/well. The pfLUC reporter construct with the luciferase gene controlled by the minimal (from -56 to +109 nt) *c-fos* mouse promoter was constructed as previously described [49]. The empty vector pcDNA3.0 was used to maintain an equal amount of DNA in all wells. Thirty hours after transfection, cells were lysed in 1X lysis buffer (6.25 mM Tris-HCl pH7.8, 10 mM DTT, 10 mM EDTA, 50% Glycerol, 5% Trition X-100). Luciferase activity was measured using kit for luciferase assay (BioVision). Activity of β-galactosidase was used to correct differences in transfection efficiency between replicates. The data is representative of at least three independent repeats. Statistical comparisons were performed using Student’s *t*-test (P < 0.02).

### Protein extraction and immunoprecipitation

Nuclear and cytoplasm proteins were extracted as described previously [15]. Briefly, the cells were scrubbed off the culture plates, resuspended in a lysis buffer (0.32 M sucrose, 2 mM MgCl_2_, 0.1 mM EDTA, 10 mM Tris–HCl, pH 7.9, 1 mM DTT, 0.4 mM PMSF), and incubated until the cells were lysed. The nuclei were purified by centrifugation through 0.5 M sucrose. Anti-RelA/p65 agarose conjugated antibodies (sc-8008 AC) was added to the precleared cytoplasm extracts (1:200 volume to volume ratio). Following overnight incubation, to control extracts without antibodies Protein G Sepharose beads were added for 2 h at +4°C.

### SDS-PAGE and Western blotting

SDS-PAGE and Western blotting were performed as described elsewhere [60]. Primary antibodies were diluted in 1X PBS with 0.1% Tween-20 (PBST). Secondary anti-mouse antibodies conjugated to HRP were diluted 1:10000 in PBST. Detection of proteins was performed using Chemidoc (BioRad) with ECL mixture (1.25 mM luminol, 0.2 mM *p*-coumaric acid, 0.01 % hydrogen peroxide in 150 mM Tris-HCl pH 8.8).
