# Erratum: Actin-binding protein alpha-actinin 4 (ACTN4) is a transcriptional co-activator of RelA/p65 sub-unit of NF-kB

**DOI:** 10.18632/oncotarget.26206

**Published:** 2018-09-28

**Authors:** Vasilisa Aksenova, Lidia Turoverova, Mikhail Khotin, Karl-Eric Magnusson, Eugene Tulchinsky, Gerry Melino, George P. Pinaev, Nickolai Barlev, Dmitri Tentler

**Affiliations:** ^1^ Institute of Cytology, Russian Academy of Sciences, Tikhoretsky av., 4, St. Petersburg, Russia; ^2^ Laboratory of Molecular Pharmacology, Saint-Petersburg Technological Institute, 26 Moskovsky Prospect, St. Petersburg, Russia; ^3^ Division of Medical Microbiology, Department of Clinical and Experimental Medicine, Linköping University, SE-581 85 Linköping, Sweden; ^4^ Department of Cancer Studies and Molecular Medicine, University of Leicester, RKCSB, LRI, Leicester, UK; ^5^ MRC Toxicology Unit, Leicester, UK; ^6^ Department of Biochemistry, University of Leicester, Lancaster Road, Leicester, UK

**This article has been corrected:** During production, the ending page number for this article was listed incorrectly. The page count has now been adjusted to show the proper pagination.

Original article: Oncotarget. 2012; 4:362-372. https://doi.org/10.18632/oncotarget.901

